# Cardioprotective potential of polyphenols rich Thraatchathi Chooranam against isoproterenol induced myocardial necrosis in experimental rats

**DOI:** 10.1186/s12906-020-03124-x

**Published:** 2020-11-23

**Authors:** Ramakrishnan Ganapathy, Anita Ramachandran, Sushmitha Basavapattana Shivalingaiah, Muhammed Bishir, Saravanan Bhojaraj, Shivashree Sridhar, Surapaneni Krishna Mohan, Vishnu Priya Veeraraghavan, Saravana Babu Chidambaram, Musthafa Mohamed Essa, M. Walid Qoronfleh

**Affiliations:** 1 Center for Animal Research, Training and Services (CAReTS), Central Inter-Disciplinary Research Facility (CIDRF), Sri Balaji Vidyapeeth (Deemed to be university), Puducherry, 607402 India; 2 International Institute of Biotechnology and Toxicology, Padappai, India; 3 Department of Pharmacology, JSS College of Pharmacy, JSS Academy of Higher Education & Research (JSS AHER), Mysuru, Karnataka 570015 India; 4 Department of Biochemistry, Panimalar Medical College Hospital & Research Institute, Varadharajapuram, Poonamallee, Chennai, 600123 India; 5 Department of Biochemistry, Saveetha Dental College, Saveetha Institute of Medical and Technical Sciences, Saveetha University, Chennai, 600077 India; 6 Central Animal Facility, JSS Academy of Higher Education & Research, Mysuru, India; 7 Department of Food Science and Nutrition, and Ageing and Dementia Research Group, College of Agricultural and Marine Sciences, Sultan Qaboos University, Muscat, Oman; 8 Research and Policy Department, World Innovation Summit for Health (WISH), Qatar Foundation, P.O. Box 5825, Doha, Qatar

**Keywords:** Thraatchathi Chooranam, Polyphenols, Antioxidants isoproterenol, Membrane enzymes, Oxidative stress, Inflammation, Apoptosis, Cardioprotective

## Abstract

**Background:**

The present study establishes the cardioprotective role of Thraatchathi Chooranam (TC), a polyherbal traditional Siddha medicine, in terms of membrane stabilizing and antioxidant properties in isoproterenol (ISO) induced myocardial necrosis model in rats.

**Methods:**

Animals were divided into six groups (*n* = 6), normal (received vehicle 0.5% CMC, p.o.), ISO control (received 0.5% CMC + ISO 120 mg/kg, b.w. s.c. twice at an interval of 48 h), standard control (received Vit-E 100 mg/kg, p.o.) + ISO, TC low and high dose (50 and 100 mg/kg p.o., respectively) + ISO, and drug control (received TC at 100 mg/kg, p.o.). At the end of experimental period, blood samples collected and plasma cardiac troponin-I (CTn-I) was measured by ELISA. Cardiac tissues were isolated, levels of membrane stabilizing enzymes, antioxidants and inflammatory markers were estimated. Gene expression of Bax, Bcl2, Caspase 3, HIF-α, TNF-α, iNOS, TRX1 and TrxR were performed by RT-PCR. Histopathological studies on cardiac tissues were conducted using hematoxylin and eosin (H&E) stain. Statistical analyses were performed by one-way ANOVA followed by Tukey’s multiple comparison as post-hoc test.

**Results:**

Administration of ISO resulted in a significant increase in plasma CTn-I, decrease in superoxide dismutase, glutathione and glutathione peroxidase; it also significantly altered membrane stabilizing enzymes like Na^+^/K^+^-ATPase, Mg^2+^-ATPase Ca^2+^-ATPase and Cathepsin D. Pretreatment with TC (50 mg/kg and 100 mg/kg) decreased CTn-I, and improved membrane stabilizing and endogenous antioxidant enzymes and decreased cathespin D level in a dose dependent manner. Histopathological examination revealed that TC improves cellular membrane integrity and decreases inflammatory cell infiltration and necrotic death.

**Conclusion:**

The present study provided a strong evidence on the protective effects of TC against ISO-induced myocardial necrosis in rats.

## Background

Cardiovascular diseases (CVDs) are more likely to occur in middle-aged individuals resulting in life and productivity loss. CVDs are a socioeconomic burden heavily pronounced in low- and middle-income countries including India where it exceeds the current global average. In the past two decades, several epidemiological studies were conducted across India, which has shown an frightening rise in number of deaths due to CVDs [1]. In countering this epidemic, there is a very urgent need to develop innovative, evidence-based research approaches rooted in traditional medicine.

Extensive experimental studies have clearly demonstrated that free radicals could play a major role in CVDs. An upsurge of free radicals would react with membrane lipids and proteins, causing peroxidation of lipid and oxidation of protein sulfhydryl groups, respectively, which leads to loss of cellular integrity due to cell membranes structural damage [2]. This loss of cellular integrity causes depletion of high-energy phosphates (mainly Adenosine triphosphate (ATP)) and subsequent malfunctioning of ion pumps as a result massive cytosolic calcium overload, which further exacerbates depletion of ATP levels and subsequently cellular dysfunction. The overproduction of reactive oxygen species (ROS) and/or reactive nitrogen species (RNS) in association with antioxidants defense system failure could invoke apoptosis in cardiomyocytes and ultimately heart failure [3]. Therefore, any antioxidants system enhancement may offer significant protection to cardiomyocytes [4, 5]. Although, numerous medical formulations have been tried and used as a therapeutic measure against cardiac failure, patients remain at very high risk of heart failure progression.

Phytochemicals are bioactive compounds found in edible and medicinal plants that possess cardioprotective, anti-lipidemic and anti-inflammatory properties. The consumption of fruits, vegetables, extracts and herbal plants rich in bioactive compounds like polyphenols, flavonoids, tannins, etc. have been shown to reduce blood pressure, inflammation, and low-density lipoprotein (LDL) oxidation, therefore aid in the prevention of CVDs. In addition, these bioactive phyto-constituents exert beneficial effect through a series of processes, such as in regulating the expression of cardiac contractile and structural proteins, preservation of high energy phosphate, regulating the calcium levels by ion channel and preventing the mitochondrial dysfunction [6]. Numerous studies have been reported the beneficial effect of variety of dietary antioxidants (mainly polyphenols) in relation to heart and other diseases as well. Hence, identification of novel bioactive compounds with antioxidants effect may possibly render substantial advantages to counteract oxidative stress induced myocardial infarction.

Siddha system of medicine is the oldest medical systems in India and mainly practiced in the southern states like Tamilnadu and Kerala. Kayakarpam or Kayakalpam is one of the special branches in Siddha medical system, which primarily focusses on rejuvenation therapies to improve innate immunity and quality longevity. Thraatchathi Chooranam (TC) is one of the herbal rejuvenating medicines which contains equal portions of 32 herbs such as *Vitis vinifera, Phoenix dactylifera, Cyperus rotundus, Piper wallichi, Santalum album, Oryza sativa, Curcuma anguistifolia, Elattaria cardamomum, Cuminum cyminum, Vetiveria zizonoides, Zingiber officinale [dried], Piper nigrum, Piper longum, Terminalia chebula, Terminalia bellarica, Embilica officinalis, Pavonia odorata, Costus speciosus, Glyzhirrizha glabra, Pavonia zeylanica*, *Tinospora cordifolia, Gmeliana asiatica, Tribulus terrestris, Plectranthus vittiviroides, Coccinium fenestratum, Nymphaea pubaecens, Syzigium aromaticum, Curcuma aromatic, Crocus sativus, Kaempferia galangal, Neliumbo nucifera and Sitramalli* [7]. Interestingly, TC is used as rejuvenator and as cardioprotective medicine in Siddha practice. In our previous studies, TC has been chemically characterized showing the rich presence of various polyphenolic compounds, constituents such as gallic acid, ellagic acid, quercetin, naringenin, and galangin. We have also reported that TC has cardioprotective properties due to the rich presence of polyphenols in it [7–10]. The present study was designed to evaluate the effects of polyphenols rich Thraatchathi Chooranam on cardiac membranes and antioxidant enzymes in isoproterenol (ISO) challenged rats. This ISO is a synthetic catecholamine, which stimulates both β1/β2-adrenergic receptors, has been reported to cause myocardial necrosis through oxy-radicals [11]. It also induces lipid peroxidation through oxy-radicals which leads to irreversible cardiac membrane damage [12]. These deleterious cascades mimics the clinical myocardial infarction injury.

## Methods

### Drugs and chemicals

The TC concoction was procured from M/s. Arogya Healthcare Pvt. Ltd., Chennai, India Alpha-tocopherol (Vit-E), isoproterenol, 2, 2′-diphyenyl-2-picrylhydrazyl and 2, 2′-azinobis-(3-ethyl-benzothiazoline-6 sulfonic acid) were purchased from Sigma Chemical Co., St. Louis, MO, USA. Tri-sodium citrate, glutathione, bovine serum albumin, sucrose, and Tris HCL were purchased from Merck. Ascorbic acid, 3,5-dinitrosalicylic acid (DNS), nitroblue tetrazolium (NBT), phenazine methosulphate (PMS), reduced glutathione, 3,5 dinitrobenzoic acid, and 2,4 dinitrophenyl hydrazine were purchased from SRL, SISCO Laboratories, Mumbai, India.

### Animals

Adult male Sprague Dawley rats (150–200 g) were used in this study. The animals were housed in an air–conditioned room at 22 ± 3 °C (RH 30–70%, 12 h - 12 h day light cycle) and fed with standard laboratory diet (Amrut Laboratory Animal Feed, Pune, India) and water ad libitum. The animals were procured from the M/s. Biogen Laboratory Animal Facility, Bengaluru, India. The Institutional Animal Ethical Committee (IAEC) at Sri Ramachandra University approved this investigation (IAEC-XXXVI/SRU/336/2013).

### Induction of myocardial necrosis

The β-adrenoceptor agonist ISO was freshly prepared in normal saline and injected subcutaneously at a dose of 120 mg/kg body weight (b.w.) into the rats on days 26 and 28 at an interval of 48 h [10].

### Experimental design

Five days after acclimatization, the rats were divided into 6 groups (*n* = 6/group). Group treatments were as follows:

- Normal control – received 0.5% carboxymethyl cellulose (CMC) + saline via subcutaneous (s.c.) injection;
- ISO control – received 0.5% CMC + ISO (120 mg/kg, b.w. s.c. twice at an interval of 48 h);
- Standard control – received Vit-E (100 mg/kg/day, oral administration-per os (p.o.)) + ISO;
- TC low dose – received TC (50 mg/kg/day, p.o.) + ISO;
- TC high dose – received TC (100 mg/kg/day, p.o.) + ISO;
- Drug control – received TC (100 mg/kg/day, p.o.)

Following pretreatment with TC and vitamin E (Vit-E) for a period of 28 days, the animals were treated with ISO on day 26 and 28 and were euthanized by carbon-dioxide (CO_2_) asphyxiation using rodent euthanasia chamber placed in necropsy room without removing animals from their home cage. This method provides a rapid, painless, stress-free death in animals. Animals were exposed to CO_2_ until complete cessation of breathing for 2–3 min. Presumed death was confirmed on careful assessment of the animal for unambiguous signs of death.

Afterwards, the hearts were excised out immediately, rinsed with ice-cold saline, blotted with filter paper, and homogenized. The heart tissue homogenates were prepared in ice-cold 10% potassium chloride and used for biochemical analyses [12]. The rat blood samples were also collected for further biochemical analysis.

### Estimation of plasma cardiac troponin I in the experimental animals

Plasma cardiac troponin I (CTnI) level was measured using an ELISA kit (Calbiotech, El Cajon, CA, USA).

### Estimation of cardiac lipid peroxidation and antioxidant profile

Tissue lipid peroxidation was measured as thiobarbituric acid reactive substances (TBARS) [13]. Reduced glutathione (GSH) was determined as described by Rahman et al. [14]*.* Superoxide dismutase (SOD) was quantified as described previously by Uikey et al. [15]. Glutathione peroxidase (GPx) was determined as per the protocol described by Weydert and Cullen method [16].

### Estimation of cardiac membrane stabilizing enzyme levels

Pellets obtained from tissue homogenate after centrifugation were re-suspended in ice-cold Tris buffer (10 mM, pH 7.4) were used for the estimation of Na^+^/K^+^-ATPase [17], Ca^2+^-ATPase and Mg^2+^-ATPase [18]. Protein content in heart tissue was estimated as described by Lowry et al. [19].

### Estimation of cardiac inflammatory marker Cathespin D activity

Cathepsin D activity was determined as per Mohammadpour et al. method [20].

### Gene expression using RT-PCR

Gene expression of Bax, Bcl2, Caspase 3, HIF-α, TNF-α, iNOS, TRX1 and TrxR were performed using heart tissue excised from all experimental groups [21]. The difference in gene expression was quantified by the Bio1D software using a gel documentation unit (Vilber Loumart, France). The primers for the genes of interest used here are mentioned in Table 1.

**Table 1:** Primers sequences

Gene	Forward sequence (5′ > 3′)	Reverse sequence (3′ > 5′)
TNF-α	GGTGATCGGTCCCAACAAGGA	CCCAGAGCCACAATTCCCTT
iNOS	CTTTACGCCACTAACAGTGGCA	AGTCATGCTTCCCATCGCTC
Bax	CTGCAGAGGATGATTGCTGA	GATCAGCTCGGGCACTTTAG
Bcl2	CCGGGAGAACAGGGTATGATAA	CCCACTCGTAGCCCCTCTG
Caspase-3	CAAGTCGATGGACTCTGGAA	GTACCATTGCGAGCTGACAT
HIF-α	CGAAGAACTCTCAGCCACAG	AGCTCGTGTCCTCAGATTCC
TRX1	TTCCCTCTGTGACAAGTATTCCAA	AGGTCGGCATGCATTTGACT
TrxR	CAACGTCCCCACAACTGTC	ATCCCGATCTGCCACTGT
β-actin	TTCTACAATGAGCTGCGTGTG	GAATCCTGTGGCATCCATGAA

### Histopathology

Heart tissues were washed with saline immediately following necropsy and then fixed in 10% buffered neutral formalin solution. After fixation, the heart tissue was processed, embedded in paraffin, sectioned (5 μm), stained with hematoxylin and eosin (H&E) then examined under a light microscope. The histo-architectural changes and photomicrographs were documented at 20x magnification for further analysis.

### Statistical analysis

Data were expressed as mean ± standard error mean (SEM). Statistical analysis was performed using GraphPad Prism, 5.0 San Diego, USA. Mean differences between groups were analyzed by one-way ANOVA followed by Tukey’s multiple comparison as post hoc test. The *p* value ≤0.05 was considered statistically significant.

## Results

### Pretreatment with TC decreased the cardiac troponin-I in ISO treated rats

**Fig. 1:**
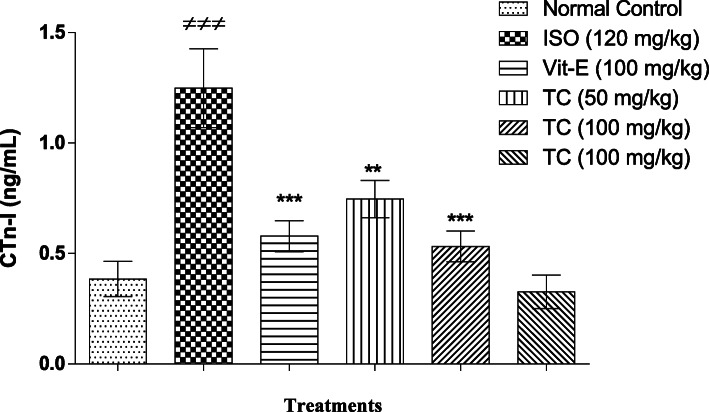
Effects of TC on plasma cardiac troponin-I in ISO administered rats. Data were expressed as mean ± SEM; (*n* = 6) and analyzed by one-way ANOVA followed by Tukey’s multiple comparison as post-hoc test. ^###^*p* < 0.001 and vs. Normal control or ^**^p < 0.01 and ^***^ *p* < 0.001 vs. ISO control group. A *p* value < 0.05 was considered as statistically significant

Cardiac troponin- I is considered as one of the important biomarkers for myocardial injury. Rats treated with ISO showed a significant increase (*p* < 0.001) in CTn-I level when compared with normal control group. Pretreatment with TC (50 and 100 mg/kg) showed a significant decrease (*p* < 0.01 and *p* < 0.001, respectively) in CTn-I level when compared with ISO control group in a dose dependent manner. The effect of TC (100 mg/kg) was comparable with Vit-E treated group (Fig. 1).

### Pretreatment with TC improved antioxidant status

**Fig. 2:**
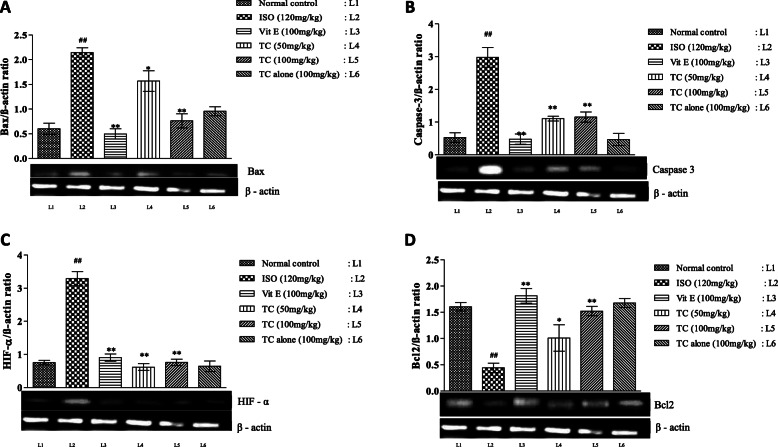
**a-d.** Dose dependent effect of TC on mRNA expression pattern of apoptotic markers Bax (2A), Caspase-3 (2B), HIF-α (2C) and Bcl2 (2D) in control and experimental groups. β-actin mRNA was used as housekeeping gene for the normalization of mRNA expressions. Quantification graphs values are expressed as mean ± SEM. ## indicates *p* < 0.01 vs normal control group; *and **- indicates *p* < 0.05 and 0.01, respectively, vs ISO group. Mean difference between the groups was analyzed using one-way ANOVA, followed by Tukey’s multiple comparison as post-hoc test. A *p* value < 0.05 was considered as statistically significant

The effect of TC on oxidative stress markers levels in control and experimental animals are summarized in Table 2. Rats treated with ISO showed significant increase (*p* < 0.01) in TBARS level and significant decrease (*p* < 0.01) in SOD, GSH and GPx levels compared with normal control group. Pretreatment with TC (50 and 100 mg/kg) decreased lipid peroxidation (LPO) and increased antioxidant levels in a dose dependent manner. The effects of TC were comparable with Vit-E. In the present study, isoproterenol group demonstrated a significant down regulation (*p* < 0.01) in the gene expression of thioredoxin (TRX1) and thioredoxin reductase (TrxR) compared to normal control group. Pretreatment with TC significantly up regulated (*p* < 0.01) TRX1 and TrxR expressions compared to ISO group (Fig. 2a and b).

**Table 2:** Effect of TC on cardiac TBARS level and antioxidant profile in ISO induced rats

Treatment	SOD units/min/mg protein	GSH nm/ g tissue	GPx nm/min/mg protein	TBARS nm/ g protein
Normal Control	9.48 ± 1.32	1.59 ± 0.30	17.17 ± 2.77	45.41 ± 3.75
Isoproterenol (120 mg/kg)	2.72 ± 0.40^##^	0.60 ± 0.10^##^	6.14 ± 0.64^##^	95.69 ± 8.54^##^
Vitamin E (100 mg/kg)	9.95 ± 1.91**	1.68 ± 0.12**	15.85 ± 0.86**	50.49 ± 3.41**
Low dose TC (50 mg/kg)	8.58 ± 1.42*	1.47 ± 0.21*	12.51 ± 1.19*	59.13 ± 2.80**
High Dose TC (100 mg/kg)	9.24 ± 0.94**	1.66 ± 0.04**	14.09 ± 0.81**	52.64 ± 2.73**
Drug Control TC (100 mg/kg)	6.93 ± 0.58	1.23 ± 0.16	13.71 ± 1.30	38.43 ± 1.78

The results are expressed as mean ± SEM (*n* = 6/group); mean differences between the groups was analyzed using one-way ANOVA with Tukey test for multiple comparison # and ## denotes *p* < 0.05 and *p* < 0.01, respectively, when compared with normal control group *and** denotes *p* < 0.05 and *p* < 0.01, respectively, when compared to isoproterenol group. *p* ≤ 0.05 is considered as statistically significant. Biostatistical analysis was performed using GraphPad Prism 5.0 Version

### Effect of cardiac membrane stabilizing enzyme levels

The effects of TC on membrane stabilizing enzymes are depicted in Table 3. The activity of Ca^2+^ ATPase was significantly increased (*p* < 0.01) while that of Na^+^/K^+^-ATPase and Mg^2+^-ATPase were significantly reduced (*p* < 0.01) in ISO group compared with normal control group. Pretreatment with TC (50 and 100 mg/kg) significantly improved membrane stabilizing enzymes such as Na^+^/K^+^-ATPase (*p* < 0.01) and Mg^2+^-ATPase (*p* < 0.01) activities and decreased the activity of Ca^2+^-ATPase (*p* < 0.01) in comparison to ISO group. Vit-E treated animals showed the reversed changes on membrane stabilizing enzymes [Ca^2+^-ATPase (*p* < 0.01); Na^+^/K^+^-ATPase (*p* < 0.01)] compared to rats administered with ISO. Interestingly, Vit-E did not produce any significant alteration in Mg^2+^-ATPase. TC alone treated group did not show any untoward changes.

**Table 3:** Effect of TC on membrane stabilizing enzymes in heart tissue normal and ISO induced rats

Treatment	Na^**+**^ / K^**+**^ ATPase (μg/min/mg protein)	Ca^**2+**^ ATPase (μg/min/mg protein)	Mg^**2+**^ ATPase (μg/min/mg protein)
Normal Control	38.49 ± 3.18	27.62 ± 1.17	29.13 ± 1.58
Isoproterenol (120 mg/kg)	15.14 ± 1.54^##^	40.74 ± 3.40^##^	17.54 ± 1.91^##^
Vitamin E (100 mg/kg)	32.39 ± 2.87**	26.64 ± 1.57**	23.30 ± 1.39
Low dose TC (50 mg/kg)	26.23 ± 2.30*	24.63 ± 1.97*	25.61 ± 1.44*
High Dose TC (100 mg/kg)	29.83 ± 1.18**	24.84 ± 2.42**	27.55 ± 2.26**
Drug Control TC (100 mg/kg)	37.36 ± 2.66	30.35 ± 2.37	22.04 ± 1.27

The results are expressed as mean ± SEM (*n* = 6/group); mean differences between the groups was analyzed using one-way ANOVA with Tukey test for multiple comparison # and ## denotes *p* < 0.05 and *p* < 0.01, respectively, when compared with normal control group *and** denotes *p* < 0.05 and *p* < 0.01, respectively, when compared to isoproterenol group. *p* ≤ 0.05 is considered as statistically significant. Biostatistical analysis was performed using GraphPad Prism 5.0 Version

### Pretreatment with TC attenuated ISO induced inflammation in cardiomyocytes

**Fig. 3:**
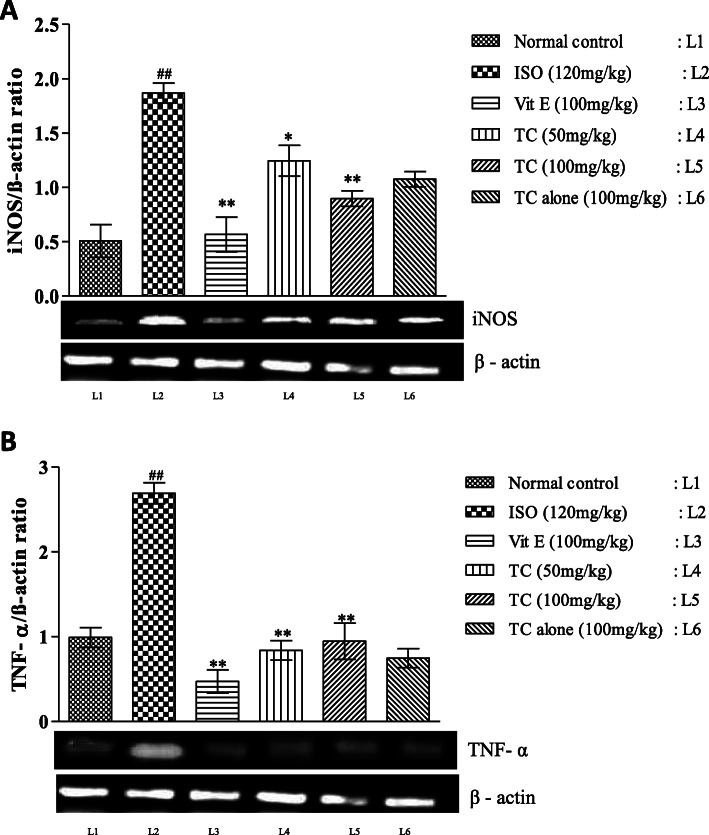
**a-b.** Dose dependent effect of TC on mRNA expression pattern of anti-inflammatory markers iNOS (3A) and TNF-α (3B) in control and experimental groups. β-actin mRNA was used as housekeeping gene for the normalization of mRNA expressions. Quantification graphs values are expressed as mean ± SEM. ## indicates *p* < 0.01 vs normal control group; *and **- indicates *p* < 0.05 and 0.01, respectively, vs ISO group. Mean difference between the groups was analyzed using one-way ANOVA, followed by Tukey’s multiple comparison as post-hoc test. A *p* value < 0.05 was considered as statistically significant

The effect of TC on cathepsin D activity is shown in Table 4. ISO groups showed a significant increase (*p* < 0.01) in cathespin D activity compared to normal control group. However, in comparison to rats administered with ISO, rats pretreated with TC and Vit-E significantly decreased cathespin D activity (*p* < 0.01). TC alone treated groups showed normal levels of inflammatory marker. Significant up regulation (*p* < 0.01) of TNF-α and iNOS expression were observed in the heart tissue of ISO group compared to normal control group. Pretreatment with TC significantly down regulated (*p* < 0.01) the TNF-α and iNOS expressions compared to ISO group (Fig. 3a and b).

**Table 4:** Effect of TC on inflammatory marker in ISO induced rats

Treatment	Cathespin D (μg/min/mg protein)
Normal Control	0.95 ± 0.08
Isoproterenol (120 mg/kg)	3.10 ± 0.35^##^
Vitamin E (100 mg/kg)	1.14 ± 0.12**
Low dose TC (50 mg/kg)	1.98 ± 0.20**
High Dose TC (100 mg/kg)	1.26 ± 0.13**
Drug Control TC (100 mg/kg)	0.95 ± 0.10

The results are expressed as mean ± SEM (*n* = 6/group); mean differences between the groups was analyzed using one-way ANOVA with Tukey test for multiple comparison # and ## denotes *p* < 0.05 and *p* < 0.01, respectively, when compared with normal control group *and** denotes *p* < 0.05 and *p* < 0.01, respectively, when compared to isoproterenol group. *p* ≤ 0.05 is considered as statistically significant. Biostatistical analysis was performed using GraphPad Prism 5.0 Version

### Pretreatment with TC mitigated ISO induced apoptosis in cardiomyocytes

**Fig. 4:**
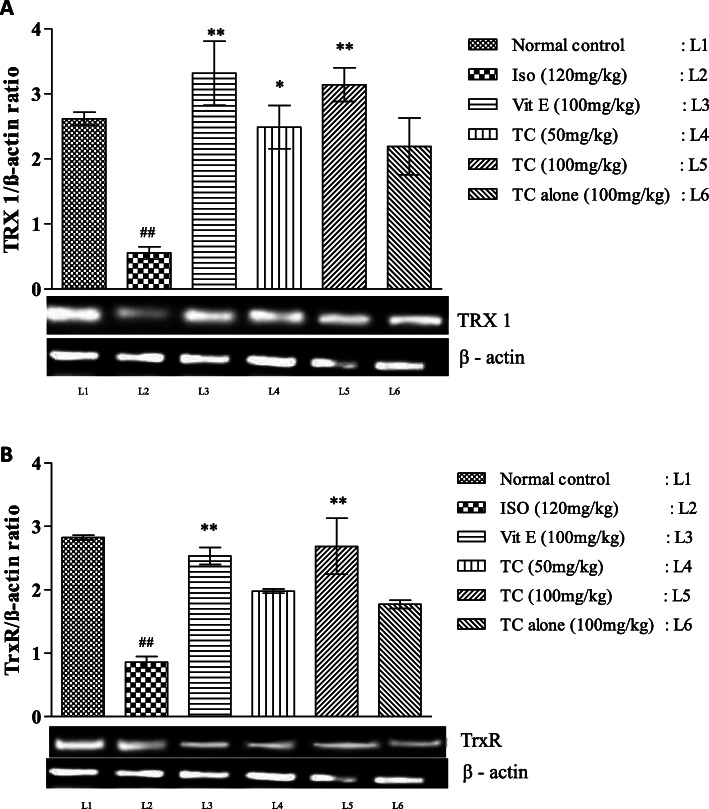
**a-b.** Effect of TC on mRNA expression of oxidative stress marker TRX1 (4A) and TrxR (4B) in cardiac muscle in control and experimental groups. β-actin mRNA was used as housekeeping gene for the normalization of mRNA expressions. Quantification graphs values are expressed as mean ± SEM. ## indicates *p* < 0.01 vs normal control group; *and **- indicates *p* < 0.05 and 0.01, respectively, vs ISO group. Mean difference between the groups was analyzed using one-way ANOVA, followed by Tukey’s multiple comparison as post-hoc test. A *p* value < 0.05 was considered as statistically significant

In the present study, administration of ISO to rats significantly up-regulated (*p* < 0.01) pro-apoptotic genes such as Bax, caspase-3, HIF-α, and down-regulated anti-apoptotic gene Bcl2 in the heart tissue compared to normal control group. TC also down regulated Bax, caspase-3 and HIF-α (*p* < 0.01) whilst up-regulating Bcl2 gene expression (*p* < 0.01) when compared to ISO group (Fig. 4a-d).

### Histopathology examination

**Fig. 5:**
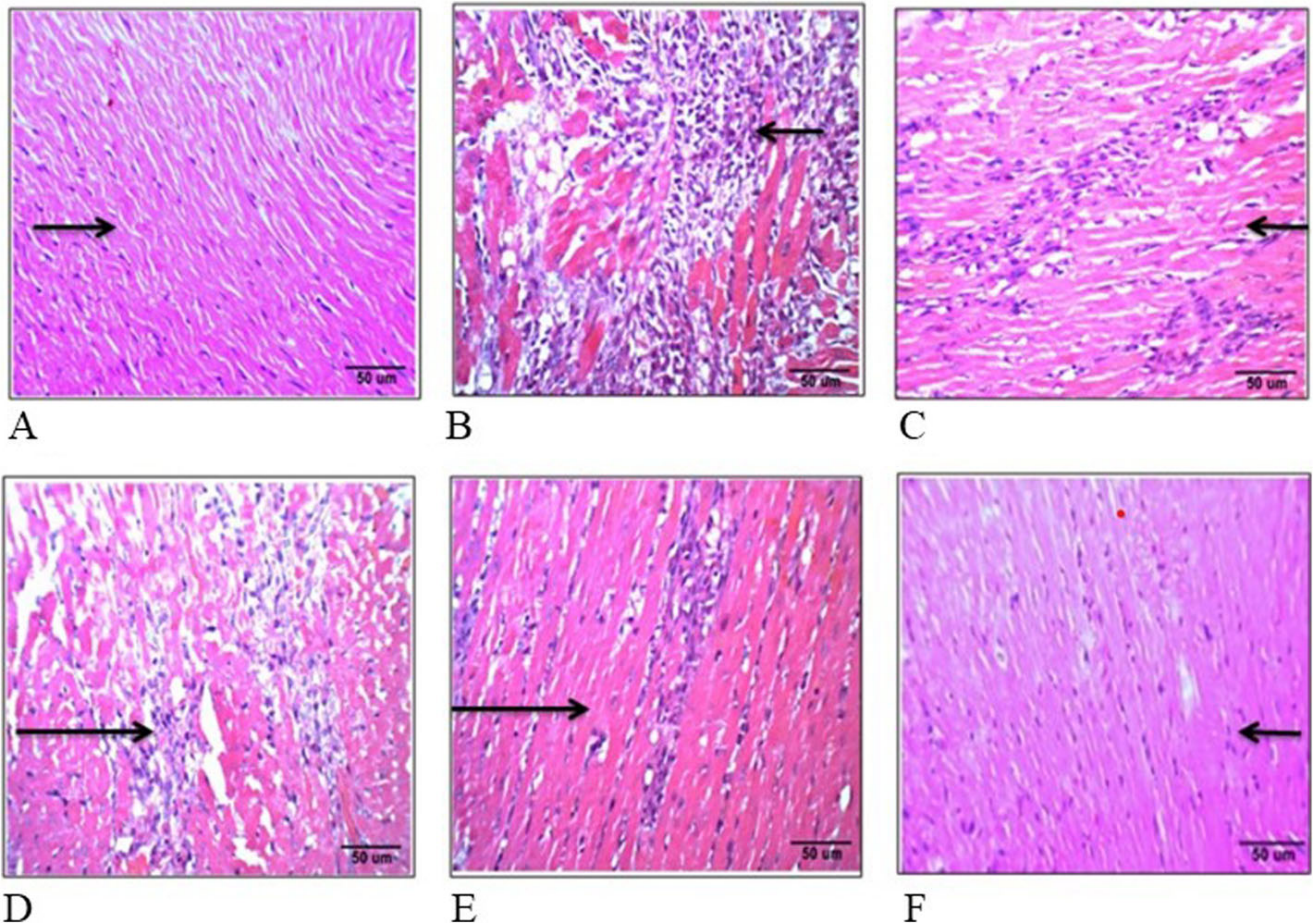
**a-f.** Effect of TC on myocardial histopathological changes in rats. Groups: 5A- Normal Control; 5B- ISO (120 mg/kg); 5C- Vit-E (100 mg/kg); 5D- TC (50 mg/kg); 5E- TC (100 mg/kg); 5F- TC alone (100 mg/kg)

The H&E staining of myocardial sections from control group showed normal cardiac muscle fibres (Fig. 5a). Myocardial sections from ISO treated rats showed severe necrosis, disorientation and swelling in cardiac muscle fibres. Moderate edema and marked infiltration of inflammatory cells were also observed as compared to control group. Low dose TC group showed moderate degeneration and necrosis of myofibres. Mild degree of edema inflammatory cell infiltration was also noted (Fig. 5d). High dose of TC showed mild focal degeneration of cardiac myofibres and mild degree of inflammatory cell infiltration (Fig. 5e). Vit-E treated rats showed mild degree of necrosis with mild degree of inflammatory cells infiltration (Fig. 5c). Drug control TC (100 mg/kg) showed no degeneration of cardiac myofibres and inflammatory cell infiltration (Fig. 5f).

## Discussion

The current investigation was undertaken to unravel the cardioprotective mechanism of TC in ISO induced myocardial necrosis rat model. The ISO administration induced myocardial necrosis and increased the cardiac injury marker CTn-I level, probably due to the surge in free (oxy) radicals. These oxy-radicals play an important role in the development of cardiovascular disease, including atherosclerosis, ischemia-reperfusion injury and stroke. Increased production of free (oxy) radicals after ischemia/reperfusion has been implicated in myocardial necrosis through complex cellular and molecular mechanisms, and it is further aggravated by impaired endogenous antioxidant defense systems. This condition favors a disruption of the redox balance, contributing to increased levels of lipid peroxides, which in turn leads to irreversible myocardial damage. Endogenous antioxidants (enzymatic SOD, CAT, GPx and non-enzymatic GSH and vitamin E) neutralize the deleterious effect of ROS through different mechanisms, including hydrogen donor, electron donor, singlet oxygen quencher, scavenging the initiating oxy radicals and binding metal ions to prevent from initiating radical formation and peroxide decomposer [22, 23].

Elevated CTn-I level is an indicator of myocardial membrane damage which causes leakage of cytosolic proteins in to blood stream [24]. Polyphenols protects cell membrane by virtue of their antioxidant and anti-inflammatory responses [25, 26]. Treatment with TC produced a dose dependent decrease CTn-I in ISO administrated rats, which indicate its protective effect on cardiac membrane integrity. Suppression of free radicals by polyphenols could be one of the major reasons for this observed protection. Similarly, we have shown earlier that TC could decrease CK-MB and LDH levels in ISO administered rats which adds further support for the beneficial action of TC on membrane stabilization [10].

In the present study, we studied the membrane stabilizing potential of TC by measuring various cardiac membrane bound enzymes like Na^+^/K^+^-ATPase, Mg^2+^-ATPase and Ca^2+^-ATPase activity in ISO treated rats. High-energy phosphates are believed to play a vital role in contraction and relaxation of cardiac muscle by maintaining normal ion levels (Ca^2+^, Na^+^, K^+^, and Mg^2+^). Functional modifications of these ion pumps could affect the normal cardiac function. High doses of ISO cause structural instability of the cell membranes, which was reflected by the lowered activity of Na^+^/K^+^-ATPase and Mg^2+^-ATPase and an increase in Ca^2+^-ATPase activity. These functional changes of ions pumps were due to altered lipid oxidation and phospholipids content in myocardium following high dose of ISO injection [27, 28]. Pretreatment with TC (50 and 100 mg/kg) significantly restored these ATPase activities. This improvement in membrane stabilization could be attributed to the prevention of the ‘SH’ group from oxidative damage by TC.

Inflammation plays a key role in all aspects of coronary diseases including initiation and progression of atherosclerotic plaque and plaque rupture, especially in recurrent thrombosis where oxidative stress is believed to play a critical role. Cathepsin D, a main inflammatory marker is involved in pathogenesis of myocardial necrosis. High dose of ISO induced necrosis resulting from increased lysosomal hydrolyses activity that was shown to be responsible for myocardial damage and infarction [29]. In this study, we observed the increased cathepsin D levels indicating the necrotic damage in myocardial membrane, which was lowered in the animals pretreated with polyphenol rich TC. Polyphenol protects lysosomal membrane from oxy radical through reduced glutathione system involvement, thereby preserving the structural and functional integrity [30]. These results tend to provide further evidence that polyphenols rich TC might have exerted its protective actions by modulating the lysosomal stability.

Further, a significant increase in the level of lipid peroxidation (LPO) and decrease in the activity of SOD, GSH and GPx were observed in ISO treated group when compared to normal control because of myocardial damage. TC (50 and 100 mg/kg) was more effective in restoring the LPO and endogenous antioxidant enzymes in a dose dependent manner. Polyphenols, by virtue of their antioxidant action are able to donate electron or hydrogen to detoxify the singlet oxygen species or peroxyl radical [31]. Hence, LPO and endogenous antioxidant activity restoration can be attributed to the high polyphenol content in TC.

In this study, we observed up regulation of myocardial Bax, HIF-α and caspase-3 gene expression and down regulation of Bcl2 gene expression in ISO group. Pretreatment with TC up regulated the expression of Bcl2 and down regulated the expression of Bax, caspase-3 and HIF-α genes. Over-stimulation of adrenergic receptors and increased contractility of myocardium consequently causes ischemia, and subsequently apoptosis [32]. TC could be able to down-regulate the pro-apoptotic gene expressions demonstrated that TC has potent anti-apoptotic property, which was in line with the previous report demonstrating TC inhibition of pro-apoptotic genes induced by H_2_O_2_ in H9c2 cells [8].

High dose of ISO injection causes functional alteration of myocardium and endothelial dysfunction, which are likely to trigger neutrophil infiltration and release of inflammatory mediators [33]. Chronic β-Adrenergic stimulation in myocardium triggers pro-inflammatory cytokine expression [34]. These changes in myocardium and endothelium were due to increased TNF-α and iNOS expressions. Polyphenol rich TC pretreatment significantly down regulated these inflammatory markers expression, which indicates its potent anti-inflammatory activity [35]. Based on the above findings, the anti-inflammatory activity and inhibition of ROS/RNS production is probably ascribed to the high phenol content in TC.

The polyherbal TC formulation has a number of medicinal ingredients with proven anti-inflammatory effects, which includes *Terminalia chebula*, *Terminalia bellarica* [36], *Piper longum*, *Emblica officinalis, Zingiber officinale* [37], *Pavonia odorata, Costus speciosus* [38], *Tinospora cordifolia* [39], and *Tribulus terrestris* [40]. Antioxidant enzymes, thioredoxin (TRX1) and thioredoxin reductase (TrxR) said to play a major role in maintaining the redox equilibrium [41]. Both TC and Vit-E treatments showed significantly up regulated (*p* < 0.01) TRX1 and TrxR expressions. Collectively, these results suggest that TC might be an adjuvant therapeutic choice for CVD due to its potent antioxidant activity. Moreover, the effect of TC in regulating the expression of inflammatory markers is another advantage besides its antioxidant capacity.

Staining of cardiac section with H&E in vehicle control rats showed normal cardiac muscle fibres. Histological observations show considerable changes like disorientation and swelling of cardiac muscle fibres, necrosis, and moderate edema with infiltration of inflammatory cell in ISO treated group. These histopathology observations along with the biochemical and molecular changes that arose established the myocardial injury. As reported earlier, these effects could be mediated through oxidative stress pathway [8, 10]. However, in Vit-E and TC (100 mg/kg) pretreated groups, such severity was not observed, signifying the protective effects.

Thus, altogether the data from this current study indicate that TC has potential membrane stabilising, anti-inflammatory and antioxidant properties, which may be due to the presence of high content of polyphenolic compounds. Further, this conclusion is consistent with the evidence presented to support the action of bioactive polyphenols and their phenolic derivatives exerting biological activity via mechanisms that can be activated by physiologically relevant concentrations [39]. Polyphenols metabolites are bioavailable and have been proposed to be even more powerful than their parent compound [39]. A recent review discussed the relevant polyphenol ligands [examples: gallic acid, ellagic acid, quercetin, and naringenin], protein targets and biochemical pathways implicated [39]. All these phytochemicals are found abundantly in the TC polyherbal formula per our initial characterization [7].

## Conclusion

Epidemiological studies suggest that polyphenol-rich intake (medicinal plants and polyphenol-enriched extracts) has high beneficial effects on multitude of cardiovascular risk factors. The mechanisms involved in the cardioprotective effects of polyphenols are varied and include antioxidant, anti-inflammatory, anti-apoptotic, anti-fibrotic, and metabolic pathways [40]. Cardioprotection by natural polyphenols has been extensively reviewed recently with a particular focus on experimental evidence and clinical outcome [41]. Some of the TC’s reported bioactive phytochemicals are gallic acid, ellagic acid, quercetin, naringenin, and galangin [8].

Thraatchathi Chooranam (TC), a polyherbal Siddha medicine from India, is rich in polyphenolic principles, exerted potent antioxidant, anti-inflammatory and anti-apoptotic properties in experimental rats challenged with isoproterenol. It is interesting to note that the cardiac membrane stabilizing effects of TC could be corroborated to potent free radical scavenging in turn lipid membrane protection. Histopathological studies also offers supportive evidence that TC reduces swelling of cardiac muscle fibres with infiltration of inflammatory cell ISO induced myocardial necrosis in rats. Taken together, the results of this study clearly demonstrates the potential cardioprotective activity of TC due to its high polyphenolic content.

In short, in this investigation several relevant, indicative markers were screened to determine the antioxidant, anti-apoptotic, anti-inflammatory as well as the membrane stabilization effects of the TC polyherbal formulation. The animal model data findings support the conclusion that TC enhanced antioxidant activities, diminished apoptotic induction, attenuated inflammatory markers levels and improved membrane stabilization. Therefore, the results of these experiments advocate further examinations to explore TC polyherbal formulation effects, active ingredients components and delineate mechanisms involved.

## Data Availability

All data generated or analyzed during this study are included in this published article.

## Abbreviations

ATP: Adenosine Tri Phosphate
CMC: Carboxymethyl cellulose
CVD: Cardiovascular diseases
CTn-I: Cardiac troponin-I
DNS: 3,5-dinitrosalicylic acid
GPx: Glutathione peroxidase
GSH: Glutathione
IAEC: Institutional Animal Ethical Committee
iNOS: Nitric Oxide Synthase
ISO: Isoproterenol
LPO: Lipid Peroxidation
PMS: Phenazine methosulphate
RNS: Reactive Nitrogen Species
ROS: Reactive Oxygen Species
SOD: Superoxide dismutase
TBARS: Thiobarbituric acid reactive substances
TC: Thraatchathi Chooranam
TNF: Tumor Necrosis Factor
TRX1: Thioredoxin
TrxR: Thioredoxin Reductase
Vit-E: Vitamin E
